# Discrepant Views of Apathy in Patients and Caregivers: the Role of Cognitive Deficits in Parkinson's Disease

**DOI:** 10.1002/mdc3.70391

**Published:** 2025-10-18

**Authors:** Giulia Funghi, Francesca Fabris, Claudia Meli, Chiara Speranza, Giuseppe Rabini, Giorgio Giulio Fumagalli, Francesca Zappini, Selene Schintu, Raffaella Di Giacopo, Stefano Tambalo, Luca Turella, Jorge Jovicich, Costanza Papagno, Alessandra Dodich

**Affiliations:** ^1^ Center for Mind/Brain Sciences (CIMeC), University of Trento Rovereto Italy; ^2^ Department of Neurology ‘Santa Maria del Carmine Hospital’, Azienda Provinciale per i Servizi Sanitari (APSS) Rovereto Italy; ^3^ Department of Physics, Molecular Biotechnology and Health Sciences University of Torino Torino Italy

**Keywords:** Parkinson's disease, apathy, discrepancy, ratings, caregivers, magnetic resonance imaging (MRI)

## Abstract

**Background:**

Apathy is a common early symptom of Parkinson's disease (PD), often co‐occurring with cognitive decline and associated with fronto‐striatal and mesocortico‐limbic dysfunctions. Discrepancies between self‐ and caregiver‐reported apathy have been preliminarily associated with cognitive impairments affecting patients’ awareness and self‐report accuracy.

**Objectives:**

This study investigates discrepancies between PD patient‐ and informant‐reported apathy in relation to the cognitive status (unimpaired‐CU vs. impaired‐CI), and explores neural correlates of apathy using magnetic resonance imaging (MRI).

**Methods:**

Apathy was assessed in 23 PD participants using self‐report (AES‐S) and informant (AES‐I) versions of the Italian Apathy Evaluation Scale. Discrepancy scores (ΔAES) were compared between groups and correlated with cognitive performance. Resting‐state fMRI examined associations between AES indices and connectivity from the bilateral nucleus accumbens, while whole‐brain structural analyses assessed associations with gray matter (GM) volume.

**Results:**

PD‐CI participants showed higher ΔAES, underestimating their apathy compared to PD‐CU. ΔAES values correlated with attentional and visuospatial functioning. Higher AES‐I scores were associated with hyperconnectivity between right nucleus accumbens, paracingulate, and medial frontal cortices. Structural analyses revealed associations between both AES‐I and ΔAES values and GM volume in the cingulate gyrus.

**Discussion:**

These findings highlight the impact of cognitive dysfunction on apathy evaluation in PD, emphasizing the importance of caregiver perspective. Neuroimaging results further validated caregiver ratings, showing an association between fronto‐striatal network changes and apathy. Further research is needed to clarify the role of such discrepancy in apathy assessment in predicting disease progression.

Apathy represents one of the most prevalent and debilitating non‐motor symptoms in Parkinson's disease (PD), with an estimated prevalence that ranges from 13.9% to 70%.^1^, ^2^ It is defined as a disorder of motivation, resulting in a diminished tendency to engage in goal‐directed behaviors, which is not attributable to cognitive decline, emotional distress, or decreased levels of consciousness.^3^, ^4^ A recent neuroscientific model of apathy in PD suggests that the deficit in goal‐directed behavior may involve more than just value‐based decision deficits. The model posits that dopamine may mediate apathy via its effects on executive function.^5^ At the neurobiological level, apathy in PD has been linked to structural and functional brain changes, particularly within fronto‐striatal and mesocorticolimbic circuits.^3^, ^6^, ^7^, ^8^ Specifically, structural imaging studies reported a reduction in gray matter volume in subcortical regions, such as the nucleus accumbens and the caudate nucleus,^9^, ^10^ as well as in cortical areas, including the frontal, insular, and posterior cingulate cortices.^10^, ^11^ Consistently, results from functional MRI studies confirmed altered patterns of fronto‐striatal and fronto‐limbic connectivity in PD compared to healthy controls.^12^, ^13^ Recent diffusion imaging studies demonstrated also global network alterations (specifically, compromised neural integration),^14^ reduced white matter connectivity,^14^ and disrupted topological organization of fronto‐striatal‐limbic circuits in apathetic PD individuals.^15^ In addition to these structural and functional brain changes (extending beyond the fronto‐subcortical regions), apathy in PD has been associated with striatal^16^ and extra‐striatal dopaminergic depletion.^17^ This dopamine depletion disrupts the brain's reward system and impacts motivation, leading to a lack of interest and reduced involvement in activities.^3^ Although striatal dopamine deficits do not directly correlate with the severity of apathy, extra‐striatal dopamine loss, particularly in regions connecting the prefrontal cortex and the limbic system, appears to be a critical factor in the development of apathy in PD.^18^

In terms of clinical assessment, apathy is usually assessed through validated questionnaires that can be administered to PD patients for self‐assessment^19^, ^20^, ^21^, ^22^ and/or to caregivers.^19^, ^23^ Notably, while some studies have reported satisfactory agreement between self‐ and informant‐reported outcomes,^24^, ^25^ others have shown significant discrepancies.^19^, ^23^, ^26^, ^27^ Among them, some have reported higher self‐reported apathy scores than caregivers,^19^, ^26^, ^27^ while others have documented lower patient apathy scores than informants.^23^, ^25^ A potential source of variability that could explain, at least in part, the observed heterogeneity of results is the cognitive status of PD patients. In this regard, Klar and colleagues^23^ suggest that one possible explanation for the discrepancy between patient and caregiver ratings is that the more cognitively impaired the patients are, the less insight they have into their apathy levels, resulting in less accurate ratings. This anosognosia would then lead to an underestimation by the patient rather than an overestimation by the caregiver. Similarly, a recent study^28^ suggests that cognitive impairment, particularly executive dysfunctions, may affect patients’ awareness of their apathetic state. This results in them reporting lower levels of apathy symptoms than their caregivers. Specifically, the authors propose that executive functioning plays a pivotal role in metacognitive processes (such as self‐monitoring and error detection) that shape the unawareness of apathetic symptoms experienced by PD individuals. Recent research has indeed emphasized the relevance of metacognition, often operationalized as the discrepancy between self‐assessment and actual performance, as a valuable framework for understanding self‐awareness impairments in neurodegenerative diseases.^29^ Although PD is not typically characterized by severe anosognosia, emerging evidence suggests that metacognitive aspects of self‐awareness may still be affected,^28^, ^30^ which could contribute to discrepancies between patient and caregiver reports. While impaired self‐awareness in PD remains poorly understood, the available preliminary evidence suggests that brain regions belonging to the salience network (e.g., the anterior cingulate cortex, the superior frontal gyrus) and the frontoparietal network contribute to unawareness in PD patients.^31^

In this context, the present study aims to investigate the discrepancy between self‐reported and caregiver‐reported levels of apathy in PD patients, focusing on the impact of cognitive status in modulating these differences. We hypothesized that cognitively impaired PD patients may significantly underestimate their levels of apathy compared to caregiver assessments. Furthermore, this study aims to examine the relationship between self‐ and caregiver‐reported apathy ratings and imaging measures, including gray matter volume and functional connectivity patterns. To this end, neuroimaging analyses were conducted both at whole‐brain level (gray matter volume) and using the nucleus accumbens as a seed (functional connectivity). Among the brain regions implicated in apathy, particular attention has been given to this region due to its pivotal role in reward processing and motivation,^32^ and its involvement in reduced goal‐directed behavior in PD.^10^, ^12^, ^33^, ^34^ Notably, a recent study by Morris and colleagues^33^ demonstrated that altered functional connectivity in the nucleus accumbens may precede the onset of apathy in PD, suggesting that early disruptions in this network may contribute to the emergence of apathetic symptoms.^12^ Previous studies, however, have shown that brain changes in PD vary according to the cognitive status, typically with predominant subcortical involvement in cognitively unimpaired patients and additional cortical alterations in cognitively impaired individuals.^35^, ^36^, ^37^, ^38^ To the best of our knowledge, no prior studies have specifically examined the neural correlates of apathy in PD as a function of cognitive impairment. Therefore, under the hypothesis that additional neural mechanisms may be involved in PD‐CI compared to PD‐CU due to more widespread neurodegeneration, we also performed exploratory connectivity analyses separately within PD subgroups stratified by cognitive profile.

## Methods

### Participants

Twenty‐three individuals (12 males; age in years: 68.02 ± 7.36; years of education: 11.96 ± 4.73; Montreal Cognitive Assessment‐MoCA^39^ adjusted score for age and years of education: 22.26 ± 3.96) diagnosed with PD according to the United Kingdom Parkinson's Disease Society Brain Bank criteria^40^ were recruited at the Centre for Neurocognitive Rehabilitation (CeRiN) of the Centre for Mind/Brain Sciences‐CIMeC (University of Trento, Italy). To be included in the study participants were required to meet the following criteria: (a) a diagnosis of idiopathic PD^40^; (b) a Hoehn and Yahr score ≤3^41^; (c) being under antiparkinsonian medication; and (d) age over 50 years. Patients with dementia or history of neuropsychiatric disorders were excluded from the study. Participants took part in the study while undergoing their usual Levodopa medication (ON state), the quantity of which was quantified through conversion to Levodopa Equivalent Daily Dose (LEDD). All participants underwent a comprehensive clinical, neurological, and neuropsychological assessment, followed by a session of structural and resting‐state functional Magnetic Resonance Imaging (MRI). No participants exhibited contraindications to MRI scanning based on their medical history and physical examination. Participants were classified as either cognitively unimpaired (CU) or cognitively impaired (CI) following the Movement Disorder Society (MDS) Level II criteria.^42^ Classification was based on performance across a neuropsychological battery encompassing the five cognitive domains recommended by the MDS Level II criteria: attention and working memory, executive function, language, memory, and visuospatial abilities. In accordance with the MDS Level II criteria, PD participants were classified as CI if they exhibited impaired performance on at least two tests, defined as either two impaired tests within the same domain or one impaired test in each of two different domains. Impairment on individual tests was determined by performance falling below the Italian normative cut‐off scores, adjusted for age, education, and sex using the regression‐based procedure proposed by Capitani and Laiacona,^43^, ^44^ which represents the standard method adopted in Italy (detailed information about the tests used and corresponding references is provided in Section S1.1 and Table S1 of the Supplementary Material). Nine PD individuals were characterized by cognitive dysfunctions in multiple cognitive domains, while three showed single‐domain alterations.

All participants provided informed consent for the study, which adhered to the ethical guidelines of the local ethics committee (University of Trento Research Ethics Committee, protocol 2019‐033) and the Declaration of Helsinki.^45^

### Behavioral Assessment

Apathy was evaluated using the Italian version of the Apathy Evaluation Scale (AES),^46^ a 18‐item questionnaire initially developed by Marin and colleagues (1991). The AES measures apathy by asking participants to indicate their level of agreement with each statement on a Likert scale ranging from 1 (“*not at all*”) to 4 (“*a lot*”), with higher scores reflecting greater severity of apathy.^47^ For this study, two versions of the AES were employed: the self‐rated (AES‐S), completed by the patient, and the informant version (AES‐I), filled out by a caregiver who cared for the patient on a routine basis and lived in the same household.

### Statistical Analyses

#### Behavioral Data

Preliminary descriptive analyses were performed in both the PD‐CI and PD‐CU groups. The distribution of continuous variables was assessed using both the Shapiro–Wilk and Kolmogorov–Smirnov tests. In addition, Q–Q plot inspection and the evaluation of skewness and kurtosis values (with corresponding Z‐scores) were used to further support decisions regarding normality. Additionally, Levene's test was used to assess homogeneity of variances between groups. Depending on the type of variable (quantitative or qualitative) and data distribution, demographic, clinical, cognitive, and social characteristics were compared between the two groups using Fisher's exact tests for categorical variables, independent samples *t*‐tests (Student's *t*‐test or Welch's *t*‐test based on variances) for normally distributed continuous variables, and Mann–Whitney *U* tests for non‐normally distributed continuous variables. Effect sizes (i.e., Cohen's d or Rank‐biserial correlation) were computed to quantify the magnitude of between‐groups differences. To control for the potential confounding effects of demographic factors, the adjusted scores derived from the Italian normative data were used for all cognitive tests. Discrepancy scores (ΔAES) were calculated by subtracting AES‐S scores from AES‐I scores ((*AES‐I)*‐(*AES‐S)*), yielding directional values. Importantly, analyses were conducted using these directional scores, not absolute values, in order to retain information about the directionality of self‐ versus informant‐report discrepancies. This approach allowed us to distinguish patients with higher AES‐I (*positive* ΔAES) from those with higher AES‐S scores (*negative* ΔAES). An independent samples *t*‐test was performed on ΔAES to test for significant differences between the two apathy assessments when comparing PD‐CU and PD‐CI individuals. Moreover, Pearson correlation coefficients were calculated to examine the relationship between discrepancy scores and all cognitive tests administered. Correlation analyses were also performed to verify the potential influence of LEDD on apathy measures (AES‐I, AES‐S, and ΔAES). Statistical analyses were performed using Jamovi 2.0.2^48^, ^49^ and JASP 0.19.3,^50^ with a level of statistical significance set at *p* < 0.05.

#### Functional Connectivity Data

Functional MRI data acquisition and pre‐processing procedures are described in the Supplementary Material (Sections S1.2 and S1.3). Head motion during resting‐state fMRI acquisition was quantified using two standard measures: mean head motion and mean frame displacement. Potential differences in these measures between PD‐CU and PD‐CI subgroups were examined to control for motion‐related artifacts.

Resting‐state functional connectivity was examined using seed‐based connectivity (SBC) analyses, focusing on the bilateral nucleus accumbens as a predefined seed region. This region was derived from the Brainnetome Atlas (https://atlas.brainnetome.org/)^51^ and selected based on converging evidence from previous studies reporting its alteration in PD patients,^52^ and specifically in PD patients with apathy.^12^, ^33^, ^34^, ^53^

Multiple regression analyses were performed on the entire PD sample to examine the relationship between resting‐state fMRI metrics and apathy metrics (AES‐I, AES‐S, and ΔAES). To further investigate the influence of cognitive status on apathy ratings, additional SBC analyses were performed within PD subgroups stratified by cognitive profiles (PD‐CU and PD‐CI) to identify connectivity patterns differentially associated with apathy scores across cognitive stages. The statistical significance level was set at *p* < 0.001 (uncorrected) voxel‐wise and *p* < 0.05 FDR cluster‐level.

Additional control fMRI analyses were performed including age, disease duration, and motor severity (UPDRS Part II and III) as covariates in the seed‐based connectivity (SBC) for each apathy measure (AES‐I, AES‐S, and AES Discrepancy) within PD participants, from the right and left nucleus accumbens.

#### Structural Data

Structural MRI data acquisition and pre‐processing procedure are described in the Supplementary Material (Sections S1.2 and S1.4). To complement the functional analyses, voxel‐based morphometry (VBM) was used to examine the relationship between gray matter (GM) volume and apathy scores. Whole‐brain multiple regression analyses were performed to identify brain regions where GM volume significantly correlated with apathy measures (AES‐I, AES‐S, and ΔAES), including the Total Intracranial Volume (TIV) as a covariate to control for confounding factors. Given the exploratory nature of this analysis, statistical significance was defined as *p* < 0.001 (uncorrected) at the voxel level. Additional control analyses were also performed using age, disease duration, and motor severity (UPDRS Part II and III) as covariates to account for their potential confounding effect.

Additionally, GM values were extracted (in subject‐specific space during pre‐processing steps) from brain areas that showed statistically significant associations with apathy metrics (AES‐I, AES‐S, and ΔAES) in the fMRI regression analysis. Correlation analyses were then performed to further investigate the relationship between GM volume in these identified regions and apathy scores in PD patients. Statistical significance was defined as *p* < 0.05 (Holm‐Bonferroni‐corrected); coordinates are reported in MNI space.

## Results

### Behavioral Data

A comparison of the demographic and clinical characteristics between PD‐CI and PD‐CU groups revealed significant differences in the Hoehn and Yahr scale score (*U* = 32.5; *p*= 0.039), the LEDD (*U* = 19.5;  *p*= 0.024), and the MoCA adjusted score (*t*(21) = −3.76; *p*= 0.001), while the two groups did not differ in terms of age, education, or sex. Significant differences were also observed in neuropsychological measures of verbal and visual memory (verbal immediate recall *t*(21) = −4.47, *p*< 0.001; verbal delayed recall *U* = 28,  *p*= 0.030; visual recall *t*(21) = −2.69;  *p*= 0.014), visuo‐perceptual and visuo‐constructional skills (Benton Facial Recognition test *t*(21) = −3.56,  *p*= 0.002; Benton Line Orientation Welch *t*‐test (9.63) = −2.28, *p*= 0.047; Rey‐Osterrieth Complex Figure Copy *U* = 24, *p*= 0.015), attention (Attentive Matrices *t*(21) = −3.54, *p*= 0.002; TMT‐B *U* = 15.0, *p*= 0.003), executive functions (TMT B‐A (*U* = 8, *p*< 0.001; Stroop time *U* = 25.0, *p*= 0.018), language (Semantic verbal Fluency *t*(21) = −3.83, *p*< 0.001; Verbs naming Welch *t*‐test (10.15) = −2.574, *p*= 0.027) and social cognition (FACE test *t*(15) = −5.90, *p*< 0.001). Table 1 presents the comparison of demographic, clinical, cognitive, and social characteristics of the two groups (PD‐CU vs PD‐CI).

**TABLE 1:** Between‐group comparisons for demographic, clinical, cognitive, and social measures

Variable	PD‐CU (n = 14)	PD‐CI (n = 9)	Statistics	Effect size
Sex (F/M)	8/6	3/6	Fisher's exact (23), *p* = 0.5	–
Age	66.6 ± 8.13	70.2 ± 5.70	*t*(21) = 1.16, *p* = 0.261	*d* = 0.494
Education	12.7 ± 4.58	10.78 ± 4.99	*t*(21) = −0.96, *p* = 0.350	*d* = −0.408
Hoehn & Yahr	1.68 ± 0.67	2.28 ± 0.44	***U* = 32.5, *p* = 0.039**	*r* = −0.484
LEDD	400.7 ± 307.5	597.3 ± 193.7	***U* = 19.5, *p* = 0.024**	*r* = −0.606
MoCA	24.23 ± 3.33	19.19 ± 2.77	***t*(21) = −3.76, *p* = 0.001**	*d* = −1.608
Digit span forward	6.24 ± 1.15	5.70 ± 1.03	*t*(21) = −1.14, *p* = 0.268	*d* = −0.486
Digit span backward	4.09 ± 0.97	3.93 ± 0.58	*t*(21) = −0.46, *p* = 0.649	*d* = −0.197
Corsi block tapping task	5.10 ± 0.93	4.68 ± 0.87	*t*(21) = −1.08, *p* = 0.291	*d* = −0.463
Rey auditory verbal learning test‐immediate recall	52.8 ± 9.06	36.51 ± 7.59	***t*(21) = −4.47, *p* < 0.001**	*d* = −1.909
Rey auditory verbal learning test‐delayed recall	11.27 ± 4.30	7.80 ± 3.15	***U* = 28, *p* = 0.030**	*r* = 0.556
Rey‐Osterrieth complex figure copy	32.1 ± 4.98	25.94 ± 6.10	***U* = 24, *p* = 0.015**	*r* = 0.619
Rey‐Osterrieth complex figure recall	19.1 ± 5.67	12.39 ± 6.16	***t*(21) = −2.69, *p* = 0.014**	*d* = −1.149
Trail making test A	29.1 ± 14.52	40.44 ± 15.20	*t*(21) = 1.80, *p* = 0.086	*d* = 0.769
Trail making test B	67.4 ± 40.2	303.78 ± 179.14	*U* = 15.0; ***p* = 0.003**	*r* = −0.762
Trail making test B‐A	39.2 ± 37.7	257.89 ± 162.11	*U* = 8; ***p* < 0.001**	*r* = −0.873
Attentive matrices	51.36 ± 6.22	40.56 ± 8.42	***t*(21) = −3.54, *p* = 0.002**	*d* = −1.513
Stroop error interference effect	−0.02 ± 0.53	3.53 ± 4.38	*U* = 40.0; *p* = 0.133	*r* = −0.365
Stroop time interference effect	14.26 ± 8.48	28.63 ± 15.87	*U* = 25; ***p* = 0.018**	*r* = −0.603
Phonetic Fluency	39.17 ± 12.00	29.29 ± 9.60	*t*(21) = −2.08, *p* = 0.050	*d* = −0.887
Semantic fluency	49.68 ± 10.34	34.82 ± 6.50	***t*(21) = −3.83, *p* < 0.001**	*d* = −1.638
Objects naming	47.3 ± 1.09	45.9 ± 2.20	*U* = 37.5; *p* = 0.089	*r* = 0.405
Verbs naming	48.44 ± 2.49	43.43 ± 5.49	***t*(10.2) = −2.574, *p* = 0.027**	*d* = −1.176
Benton line orientation judgment task	25.29 ± 2.23	20.78 ± 5.65	***t*(9.6) = −2.28, *p* = 0.047**	*d* = −1.049
Benton facial recognition test	48.43 ± 3.67	43.0 ± 3.39	***t*(21) = −3.56, *p* = 0.002**	*d* = −1.521
Ek60 global score	50.2 ± 5.93	46.85 ± 6.60	*t*(21) = −1.27, *p* = 0.219	*d* = −0.541
FACE	30.45 ± 3.21	21.04 ± 2.31	***t*(15) = −5.90, *p* < 0.001**	*d* = −3.141
AES–self	31.14 ± 6.36	30.11 ± 8.54	*t*(21) = −0.412, *p* = 0.685	*d* = −0.176
AES–informant	27.85 ± 6.63	35.55 ± 9.68	*U* = 31, *p* = 0.070	*r* = −0.47
ΔAES	−2.54 ± 8.51	5.44 ± 8.09	***t*(20) = 2.206, *p* = 0.039**	*d* = 0.956

*Note*: Adjusted scores based on Italian normative data, accounting for age and education (and sex where applicable). Data represented as mean ± standard deviation. Effect sizes (Cohen's d or Rank‐biserial correlation) are reported. Group comparisons were conducted using independent samples *t*‐tests, Welch's *t*‐tests, or Mann–Whitney *U* tests, depending on the distributional characteristics of each variable. Bold values indicate statistically significant results (*p* < 0.05).

Abbreviations: Ek60, Ekman 60‐faces test; FACE, Facial complex expressions test; LEDD, Levodopa equivalent daily dose; MoCA, Montreal cognitive assessment; Ratio‐B/F, Ratio between digit span forward and digit span backward; RAVLT, Rey auditory verbal learning test; SET, story‐based empathy task.

**Figure 1:**
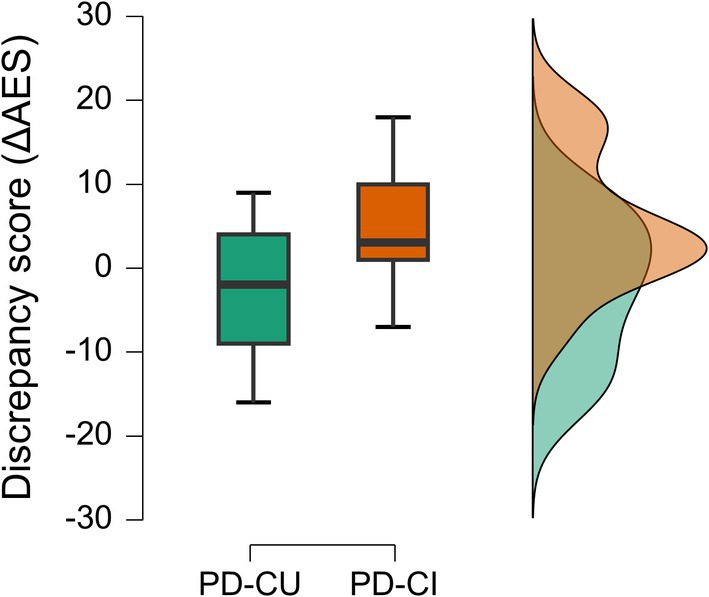
Comparison of apathy evaluation scale (AES) discrepancies (AES‐I–AES‐S) in cognitive unimpaired (PD‐CU, in green) and cognitive impaired (PD‐CI, in orange) PD patients

All PD participants enrolled in the study completed the AES‐S and 22 caregivers filled out the AES‐I questionnaire. Preliminary statistical analyses, based on Z‐scores and box plot visualization, confirmed the absence of outliers on the apathy metrics. The comparison of the ΔAES between the two groups (PD‐CU vs PD‐CI) yielded a statistically significant difference (*t*(20) = 2.21, *p*= 0.039). In particular, PD‐CI individuals exhibited positive discrepancy values (ΔAES: 5.44 ± 8.09), with caregivers reporting higher levels of apathy when compared to PD individuals’ self‐report, while PD‐CU showed an effect in the opposite direction (ΔAES: −2.54 ± 8.51), with patients reporting higher apathy levels than caregivers, although of a smaller magnitude (Fig. 1).

A significant correlation was observed between discrepancy scores and attentive and visuo‐spatial performances. In particular, the results indicated a positive correlation between discrepancy scores and longer completion times for the TMT‐A (*r*_p_ = 0.468, *p* = 0.028) and with poorer performance on the Benton Line Orientation Judgment Task (*r*_s_ = −0.47, *p* = 0.027) (Fig. S1). No further significant correlations were identified with cognitive performance or LEDD.

### Functional Connectivity Data

**Figure 2:**
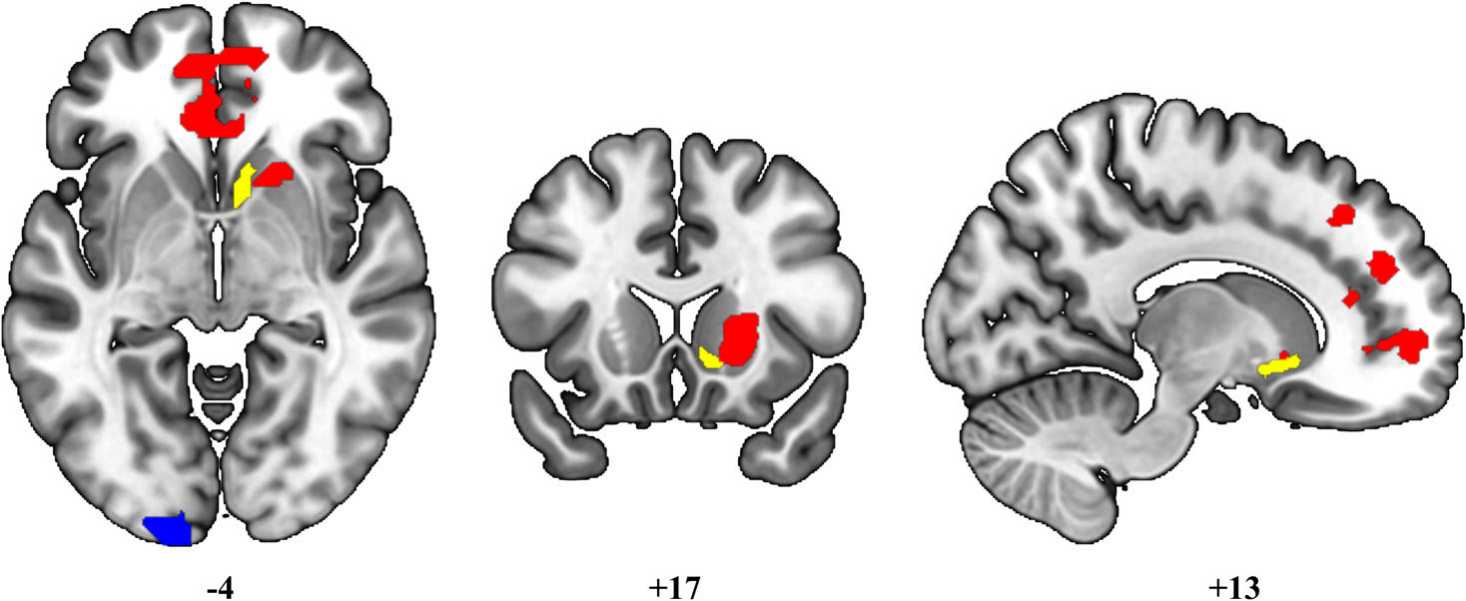
Seed‐based FC results‐regression analysis with total sample and AES‐I scores, seeding from the right nucleus accumbens. Red color indicates functional hyperconnectivity between cortical and subcortical clusters and the right nucleus accumbens (marked in yellow). Blue color indicates functional hypoconnectivity between the left occipital pole and the right nucleus accumbens (in yellow). Statistical significance level: *p* < 0.05 FDR‐corrected. Images are displayed in neurological convention.

No significant differences emerged between PD‐CI and PD‐CU subgroups in either mean head movement (mean ± st.dev: 0.8 ± 0.4, *U* = 56, *p* = 0.69) or mean frame displacement (mean ± st.dev: 0.3 ± 0.1, *U* = 59, *p*= 0.83). In the regression analysis using AES‐I scores as a measure of apathy in the whole PD sample, significant associations emerged between apathy severity and altered functional connectivity patterns seeded from the right nucleus accumbens. Specifically, higher caregiver‐rated apathy scores were associated with a hyperconnectivity pattern (depicted in red in Fig. 2) between the right nucleus accumbens and the anterior division of the cingulate gyrus (x: −04, y: +34, z: −02; p‐FDR = 0.00003), the right paracingulate gyrus (x: +10, y:+46, z: +26; p‐FDR = 0.00003), the right middle frontal gyrus (x: +16, y: +30, z: +42; p‐FDR = 0.02) and the right putamen (x: +22, y: +18, z: +04; p‐FDR = 0.02). Additionally, a significant pattern of hypoconnectivity (depicted in blue in Fig. 2) was found between the right nucleus accumbens and the left occipital pole (x: −36, y: −90, z: −16; p‐FDR = 0.00003) (Fig. S2). No significant results were found when seeding from the left nucleus accumbens. Also, no significant results were found when using AES‐S and ΔAES as variables of interest.

**Figure 3:**
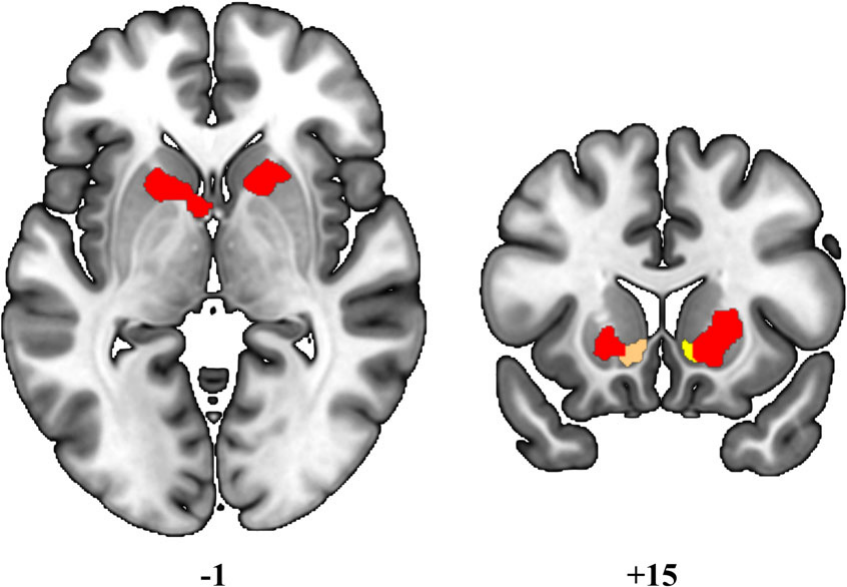
Seed‐based FC results‐regression analysis with PD‐CU and AES‐I scores, seeding from right and left nucleus accumbens. Red color indicates functional hyperconnectivity between the right nucleus accumbens (in yellow) and the subcortical cluster. Green color indicates functional hyperconnectivity between the left nucleus accumbens (in orange) and the posterior cingulate gyrus. Statistical significance level: *p*< 0.05 FDR‐corrected. Images are displayed in neurological convention.

The explorative seed‐to‐voxel regression analyses examining the relationship between functional connectivity and AES‐I scores, stratifying PD individuals by cognitive profile, showed significant results seeding from the bilateral nucleus accumbens for both PD‐CU and PD‐CI groups. In particular, in PD‐CU subjects a hyperconnectivity pattern emerged at the subcortical level between the right nucleus accumbens and both the right putamen (x: +12, y: +14, z: −06; p‐FDR = 0.0004) and the left thalamus (x: −02, y: +02, z: +06; p‐FDR = 0.0001), as well as between the left nucleus accumbens and the posterior cingulate gyrus (x: +02, y: −40, z: +08; p‐FDR = 0.003) (Fig. 3). In addition, the results indicated a functional hypoconnectivity between the left nucleus accumbens and the right occipital pole (x: +06, y: −96, z: +02; p‐FDR = 0.034).

**Figure 4:**
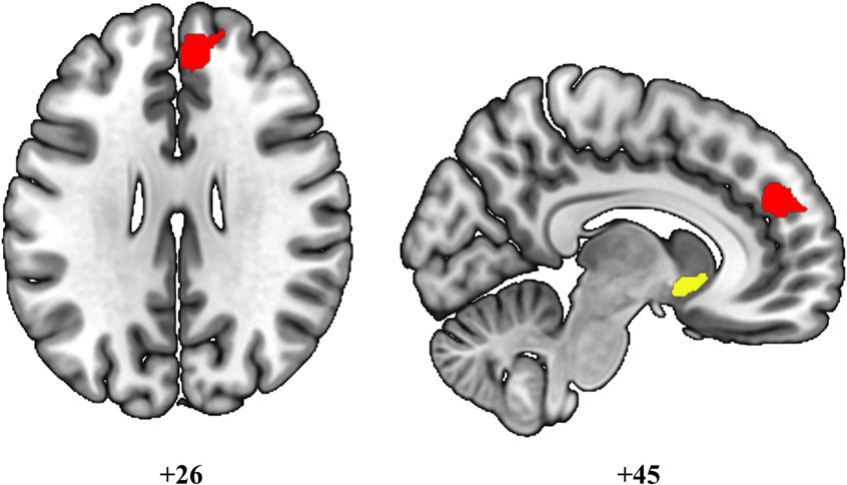
Seed‐based FC results‐regression analyses with PD‐CI and AES‐I, seeding from the right nucleus accumbens. Red color indicates functional hyperconnectivity between the right nucleus accumbens (in yellow) and the right paracingulate gyrus. The figure does not show the hypoconnectivity found between the left nucleus accumbens and the occipital cluster. Statistical significance level: *p*< 0.05 FDR‐corrected. Images are displayed in neurological convention.

On the other hand, PD‐CI subjects showed a more cortical pattern of hyperconnectivity between the right nucleus accumbens and the right paracingulate gyrus (x: +10, y: +44, z: +26; p‐FDR = 0.001) (Fig. 4). Hypoconnectivity was found between the left nucleus accumbens and the left temporal occipital fusiform cortex (x: −20, y: −60, z: −14; p‐FDR = 0.0129).

Results of the additional control fMRI analyses are reported in Supplementary Material Section 2.1 and Table S2.

### Structural Data

Whole‐brain VBM regression analysis between GM volumes and both AES‐S, AES‐I, and ΔAES values showed no significant FDR‐corrected results at the whole‐brain level. On the other hand, the ROI‐based VBM regression analysis demonstrated a statistically significant negative correlation between AES‐I scores and gray matter volume in the cingulate gyrus (x: −4, y: +34, z: −2, p‐_Holm‐Bonferroni_ = 0.014, *t*‐value 2.371), providing further evidence for the relevance of this region in the structural underpinnings of apathy in PD patients (Fig. S3). The same brain area also emerged using the ΔAES scores (p‐_Holm‐Bonferroni_ = 0.019, *t*‐value 2.416).

Results of the additional control VBM analyses are reported in Supplementary Material Section 2.2.

## Discussion

The present study aimed to investigate the impact of cognitive status on apathy assessment by comparing self‐reported and informant‐reported apathy levels in PD patients with and without cognitive impairment. Additionally, the study explored the relationship between apathy measures (AES‐I, AES‐S, and ΔAES) and functional connectivity patterns. To complement the functional connectivity findings, VBM analyses were performed to investigate structural brain changes associated with AES scores, providing a comprehensive understanding of the neurobiological correlates of apathy in PD.

The behavioral findings revealed significant differences between self‐ and informant‐reported apathy levels when stratifying the sample by patients’ cognitive profile (PD‐CU vs PD‐CI). Specifically, caregivers of PD‐CI patients reported significantly higher apathy levels compared to the patients’ self‐assessments, whereas a trend in the opposite direction (although of smaller magnitude) was found in PD‐CU group, with patients reporting slightly higher apathy levels than caregivers. These findings align with prior research on apathy in PD, which has reported a possible lack of agreement between informant‐ and self‐reported apathy evaluations.^26^, ^27^, ^28^ The possible factors contributing to these discrepancies are still a matter of debate in the literature. Some studies have reported higher self‐reported apathy compared to caregiver ratings,^19^, ^26^, ^27^ potentially reflecting overestimation of the symptom by patients^19^ or, conversely, its underestimation by caregivers.^26^, ^27^ In contrast, our findings (greater apathy rating from informants in the PD‐CI group) suggest that cognitive status may play a critical role in shaping these discrepancies, and may help to explain the variability observed in previous research.^23^ Consistently, Klar and colleagues^23^ also reported caregivers rating apathy higher than the patients themselves, and highlighted the role of both caregiver burden and cognitive status in shaping these differences. However, their analysis did not stratify the sample by cognitive profile, limiting the ability to isolate the effect of cognition. By directly comparing cognitively preserved and impaired subgroups, our findings provide further granularity, showing that such discrepancies are primarily evident in cognitively impaired patients. Specifically, cognitive impairment may hinder patients’ ability to accurately evaluate their own condition, potentially leading to underreporting of symptoms. This hypothesis is consistent with the existing literature highlighting the association between apathy, discrepancy in self‐reports, and cognitive deficits in PD.^25^, ^27^, ^28^ Interestingly, the discrepancy between self‐ and informant‐assessment of apathy was significantly associated with attentional and visuo‐spatial alterations. This is an intriguing finding, which partially diverges from previous literature that predominantly links the presence of apathy to executive dysfunction in PD.^1^ On the one hand, it may suggest that distinct cognitive mechanisms underlie apathy itself and the awareness of being apathetic.^54^ On the other hand, it is plausible to hypothesize that discrepancies in apathy assessment and attentional and visuospatial impairments may all represent early indicators of an unfavorable clinical course. This hypothesis is supported by prior literature showing an increased risk of progression associated with this cognitive phenotype.^55^ Overall, these results might support the emerging evidence linking anosognosia for neuropsychiatric disturbances to a higher risk of disease progression in PD,^56^ although more longitudinal studies are needed to confirm this hypothesis.

Our fMRI findings revealed a positive association between caregiver‐rated apathy scores and increased functional connectivity between the right nucleus accumbens and the anterior cingulate gyrus, the paracingulate gyrus, the middle frontal gyrus, and the putamen. Among these, the anterior cingulate cortex plays a pivotal role in the motivational circuit, integrating cognitive, emotional, and motivational processes, underscoring its centrality in apathy pathophysiology.^57^, ^58^ Remarkably, higher functional connectivity between the nucleus accumbens and the anterior cingulate cortex has been reported in PD patients who subsequently presented with clinically relevant apathetic symptoms.^33^ Additionally, our structural MRI findings showed a significant correlation between AES‐I scores and reduced gray matter volume in the left cingulate gyrus, reinforcing the relevance of this area in PD alterations of motivation‐related behaviors.^57^, ^59^ Notably, also the discrepancy score correlated with GM volume in this brain region, an area that has been previously reported to be implicated in self‐awareness in PD.^31^

Our findings also revealed functional hyperconnectivity between the nucleus accumbens and the middle frontal gyrus. These results are in contrast with those reported by Zhang et al,^60^ who instead observed a prefrontal hypoconnectivity pattern related to apathy severity in PD patients. The mismatch might be explained by differences in the clinical characteristics of participants (i.e., early vs more advanced disease stages). While hypoconnectivity is frequently reported in later disease stages, the hyperconnectivity pattern observed here may represent an early compensatory or maladaptive response.^12^ Our fMRI results also indicated an association between occipital functional hypoconnectivity and higher AES‐I scores, which might be interpreted as an early signature of degeneration.^61^, ^62^

In conclusion, this study underscores the significance of discrepancy measures (ΔAES) and caregiver‐reported apathy assessments (AES‐I) in the context of PD, particularly for patients with cognitive impairment who may experience anosognosia. A higher discrepancy between self‐ and caregiver‐reported apathy was observed in the PD‐CI group, with discrepancy scores correlating with attentional and visuo‐spatial alterations. These findings are further validated at the neurobiological level by the MRI results, which demonstrated an association between caregiver‐only apathy ratings and changes in the fronto‐striatal network. Conversely, no significant neuroimaging correlates were found in relation to self‐reported apathy (AES‐S). This null finding further highlights possible limitations of relying solely on self‐assessment in this population when cognitive impairment is present and reinforces the added value of informant‐based evaluations when investigating the neural substrates of apathy in PD. Collectively, our results on the neural correlates of apathy in PD are consistent with those reported by Skidmore and colleagues,^63^ which support the use of resting fMRI as a means to evaluate neuropsychiatric states (e.g., apathy and depression) in PD.

A limitation of the study is the modest sample size, which may limit the generalizability of our findings, particularly when exploring the neural substrates in PD‐CI and PD‐CU subgroups separately. These results should therefore be interpreted with caution and considered exploratory. In addition, the cross‐sectional design of the study prevented us from examining the progression of apathy and changes in self‐awareness over time, also in relation to cognitive decline. Future longitudinal research is required to clarify these temporal dynamics. Another limitation concerns the reliance on self‐ and informant‐report questionnaires to assess apathy. While these are commonly used, they may be influenced by responder biases or caregiver burden^23^ and they may not fully capture the multidimensional nature of the construct. While informants ratings may offer valuable insight in cases of reduced patient awareness, it is important to consider that caregivers burden can also bias informant reports, potentially leading to the overestimation of symptoms.^23^ These considerations should be taken into account when interpreting informant‐based assessments in clinical contexts, and future studies employing multimodal assessments and larger, longitudinal samples will be critical in validating and expanding upon the present findings. Overall, the study highlights the importance of adopting a multi‐informant approach to obtain a more comprehensive understanding of apathy in PD and supports the use of the AES‐I in clinical practice, even at the stage of mild cognitive impairment, thus in the presence of cognitive decline without impairment in daily functioning.^64^

## Author Roles

(1) Research Project: A. Conception, B. Organization, C. Execution; (2) Statistical Analysis: A. Design, B. Execution, C. Review and Critique; (3) Manuscript Preparation: A. Writing of the first draft, B. Review and Critique.

Giu.F.: 1A, 1B, 1C, 2A, 2B, 3A, 3B.

F.F.: 1A, 1C, 2A, 2B, 3A.

Gio.F.: 1A, 1C, 3B.

C.M.: 1C, 1B, 2C, 3B.

C.S.: 1C, 2A, 2B, 3A, 3B.

S.T.: 1C, 2C, 3B.

F.Z.: 1C, 3B.

S.S.: 1B, 3B.

R.D.G.: 1C, 3B.

L.T.: 1A, 1B, 1C, 2A, 2C, 3A, 3B.

J.J.: 1C, 1B, 2C, 3B.

C.P.: 1C, 1B, 2C, 3B.

A.D.: 1A, 1B, 1C, 2A, 2C, 3A, 3B.

## Disclosures

**Ethical Compliance Statement:** All procedures were performed in compliance with relevant laws and institutional guidelines, and were approved by the Ethics Committee of the University of Trento (N. prot. 2019‐033). The study was conducted in accordance with the Declaration of Helsinki of the World Medical Association: Ethical principles for medical research involving human subjects. Written informed consent for participation in the study, including the possibility of publication of the data obtained in the study, was obtained from all participants, respecting the right to privacy of human subjects. All authors confirm that they read the Journal's position on issues involved in ethical publication and affirm that this work is consistent with those guidelines.

**Funding Sources and Conflicts of Interest:** This study was supported by the Fondazione Cassa di Risparmio di Trento e Rovereto (CARITRO) project “Tango. Una terapia complementare per la malattia di Parkinson.” The project was also supported by both the Assessorato alle Politiche Sociali del Comune di Rovereto (Trento, Italy) and the Associazione Parkinson Trento. The authors declare that there are no conflicts of interest relevant to this work.

**Financial Disclosures for the Previous 12 Months:** The authors declare that there are no additional disclosures to report.

## Supporting information


**Figure S1.** Scatterplot with regression lines showing the relationship between the ΔAES score and cognitive variables. (A) Trail making test–A. (B) Benton Line Orientation Judgment Task.

**Figure S2.** Scatter plots showing the relationship between AES‐I scores and mean Fisher z‐transformed connectivity values extracted from significant clusters (seeded from the Right Nucleus Accumbens) in PD participants. Connectivity values represent the mean across all voxels within each ROI mask. Each dot corresponds to one subject and is color‐coded according to cognitive status (blue = PD‐CI, red = PD‐CU).

**Figure S3.** Scatter plot showing the relationship between AES‐I scores and regional gray matter volume extracted from significant cluster in PD participants. Gray matter volume values represent the mean across all voxels within the ROI mask. Each dot corresponds to one subject and is color‐coded according to cognitive status (blue = PD‐CI, red = PD‐CU).

**TABLE S1.** Neuropsychological tests, corresponding Italian normative references, and cut‐off scores adjusted for demographic variables.
**TABLE S2.** Regions with significant associations between AES‐I scores and right nucleus accumbens connectivity in PD (controlling for age, disease duration, and motor severity).

## Data Availability

The data that support the findings of this study are available from the corresponding author upon reasonable request.
