# Supplementary figures and images for: Discrepant Views of Apathy in Patients and Caregivers: the Role of Cognitive Deficits in Parkinson's Disease
Source: Mov Disord Clin Pract. 2025 Oct 18;13(4):923–32. doi: 10.1002/mdc3.70391 (PMC13071371; doi:10.1002/mdc3.70391)

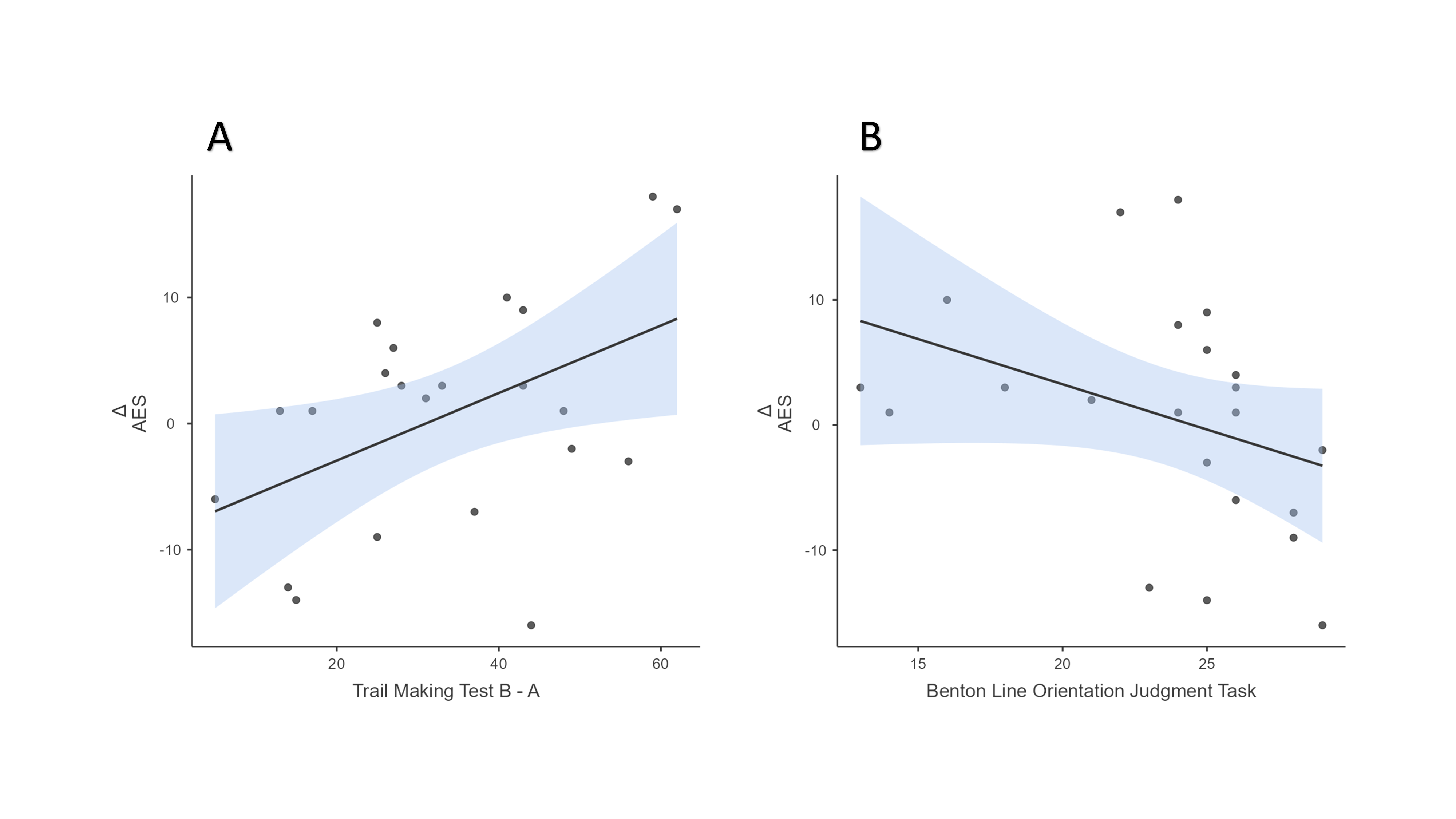

Supplement: Supplementary file 1 — Figure S1. Scatterplot with regression lines showing the relationship between the ΔAES score and cognitive variables. (A) Trail making test–A. (B) Benton Line Orientation Judgment Task. [file MDC3-13-923-s001.tiff]

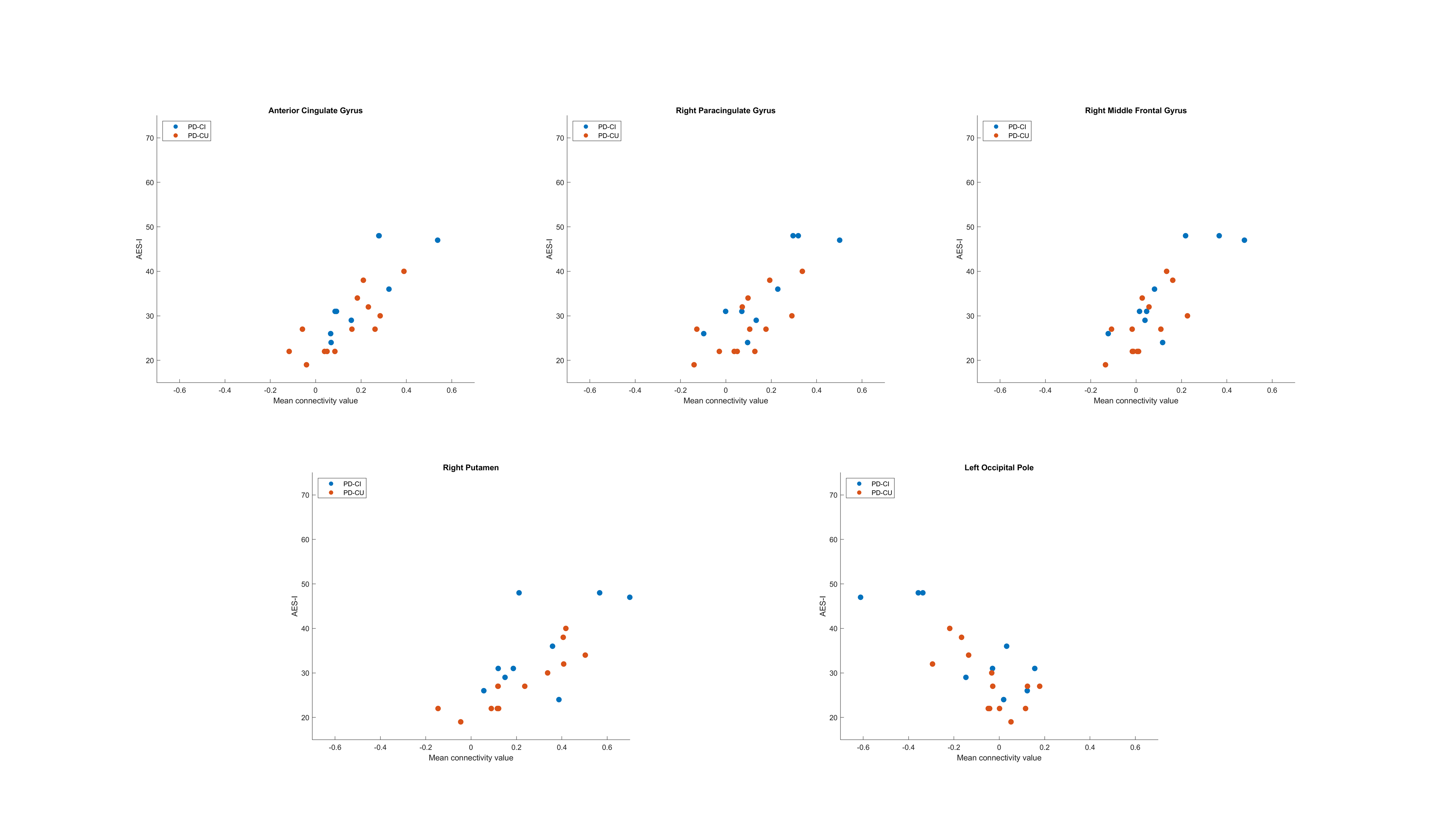

Supplement: Supplementary file 2 — Figure S2. Scatter plots showing the relationship between AES‐I scores and mean Fisher z‐transformed connectivity values extracted from significant clusters (seeded from the Right Nucleus Accumbens) in PD participants. Connectivity values represent the mean across all voxels within each ROI mask. Each dot corresponds to one subject and is color‐coded according to cognitive status (blue = PD‐CI, red = PD‐CU). [file MDC3-13-923-s002.tiff]

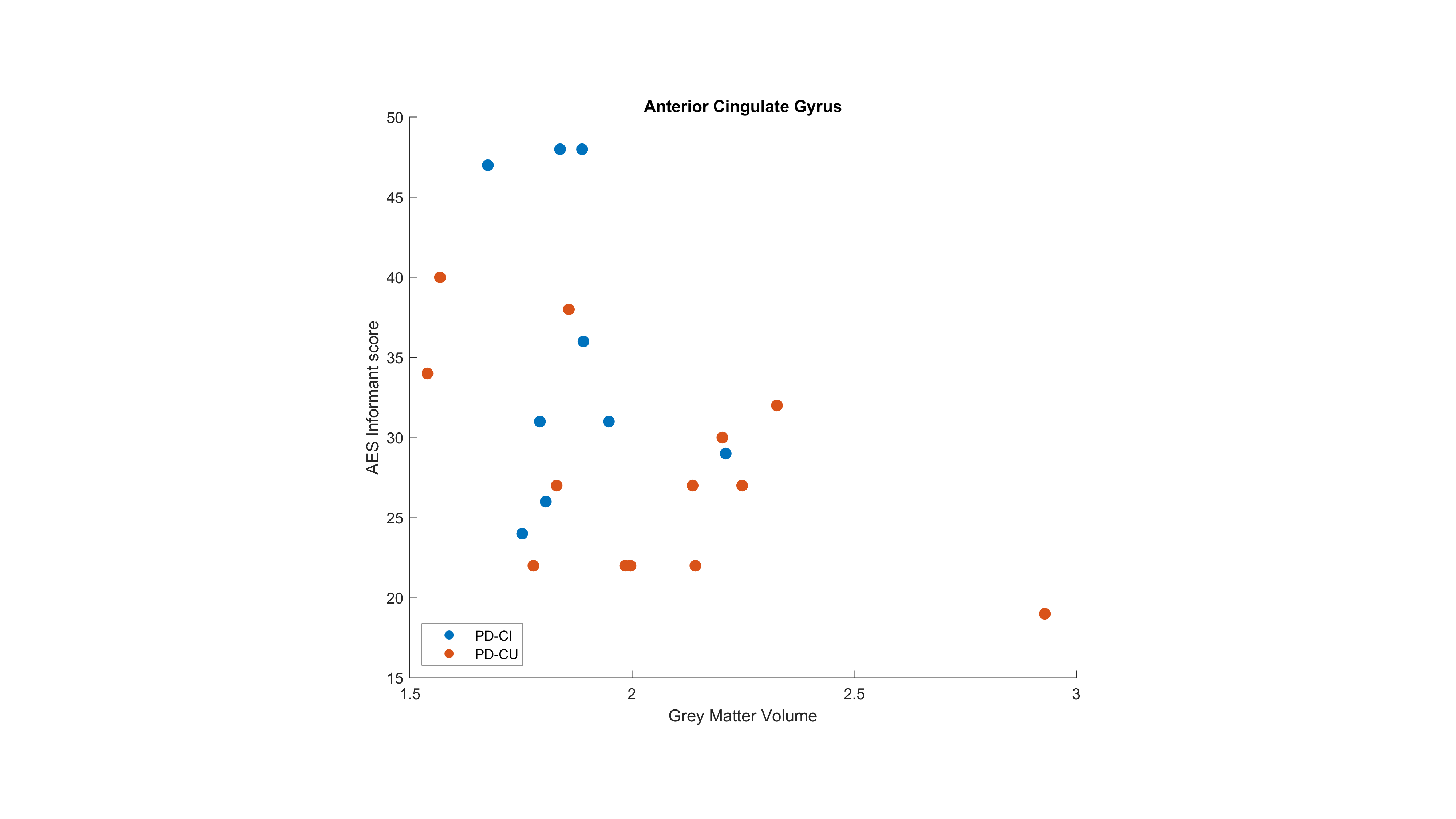

Supplement: Supplementary file 3 — Figure S3. Scatter plot showing the relationship between AES‐I scores and regional gray matter volume extracted from significant cluster in PD participants. Gray matter volume values represent the mean across all voxels within the ROI mask. Each dot corresponds to one subject and is color‐coded according to cognitive status (blue = PD‐CI, red = PD‐CU). [file MDC3-13-923-s003.tiff]
