# Supplementary material for: Discrepant Views of Apathy in Patients and Caregivers: the Role of Cognitive Deficits in Parkinson's Disease
Source: Mov Disord Clin Pract. 2025 Oct 18;13(4):923–32. doi: 10.1002/mdc3.70391 (PMC13071371; doi:10.1002/mdc3.70391)
Supplement: Supplementary file 4 — TABLE S1. Neuropsychological tests, corresponding Italian normative references, and cut‐off scores adjusted for demographic variables. TABLE S2. Regions with significant associations between AES‐I scores and right nucleus accumbens connectivity in PD (controlling for age, disease duration, and motor severity). [file MDC3-13-923-s004.docx]

**Supplementary Material**

*S1.1 Neuropsychological assessment*

The assessment of ***attention and working memory*** was conducted through the Digit Span Forward and Backward tasks (Monaco et al. 2013), the Corsi Block Tapping Test (Monaco et al. 2013), and the Attentive Matrices (Spinnler and Tognoni 1987). The delayed recall of the Rey-Osterrieth Complex Figure Recall (Caffarra et al. 2002a) and the Rey Auditory Verbal Learning Test (Carlesimo et al. 1996) were employed to evaluate long-term ***memory***. ***Executive functions*** were examined through the administration of the Stroop Test (Caffarra, Vezzadini, et al., 2002), the Trail Making Test (TMT) (Giovagnoli et al. 1996), and phonemic fluency (Carlesimo et al. 1996). ***Language*** was evaluated using semantic fluency (Zarino et al. 2014), and both object (Catricalà et al. 2013) and action (Papagno et al. 2020) naming tests. ***Visuospatial, visuo-perceptive and visuo-constructive abilities*** were assessed through the Benton Line Orientation Judgment Task (Benton, Varney, and Hamsher 1978), the Benton Facial Recognition Tests (Benton et al. 1983), and the Rey-Osterrieth Complex Figure Copy (Caffarra et al. 2002a). The performance on these tasks was used to classify patients in PD-CU (n=14) and PD-CI (n=9), based on level II diagnostic criteria for mild cognitive impairment (MCI) in PD (Litvan et al. 2012). Besides, ***social cognition*** was also evaluated, using the Ekman-60 Faces Test (Dodich et al. 2014), and the Facial Complex Expressions Test (Terruzzi et al. 2023).

***Table S1. Neuropsychological tests, corresponding Italian normative references, and cut-off scores adjusted for demographic variables.***

| ***Cognitive Tests*** | ***Cut-off score*** |
| --- | --- |
|  |  |
| ***Screening*** |  |
| Montreal Cognitive Assessment Mo.C.A (0-30) *(Conti et al., 2015)* | < 17.363 |
| ***Attention and working memory*** |  |
| Digit Span *(Monaco et al., 2013)* |  |
| - Forward (0-9) | < 4.26 |
| - Backward (0-8) | < 2.65 |
| Corsi Block Tapping Test (0-9) *(Monaco et al., 2013)* | < 3.46 |
| ***Memory*** |  |
| Rey Auditory Verbal Learning Test Delayed Recall (*Carlesimo et al., 1996*)  Rey-Osterrieth Complex Figure Recall (*Caffarra et al., 2002a*) | ≤ 4.68  ≤ 9.47 |
| ***Executive* *functions*** |  |
| Matrici Attentive (0-60) (*Spinnler & Tognoni, 1987*) | < 31 |
| Trail Making Test (*Giovagnoli et al., 1996*) |  |
| - Parte A | ≥ 94 |
| - Parte B | ≥ 282 |
| - Parte (B-A) | ≥ 187 |
| Stroop Test *(Caffarra, Vezzadini, et al., 2002)* |  |
| - Error Interference Effect | ≥ 4.24 |
| - Time Interference Effect | ≥ 36.92 |
| Phonemic Fluency *(Carlesimo et al., 1996)* | ≤ 17.35 |
| ***Language*** |  |
| Objects naming test (0-48) *(Catricalà et al., 2013)* | ≤ 41.48 |
| Actions naming test (0-50) (*Papagno et al., 2020)* | ≤ 36.86 |
| Semantic Fluency (*Zarino et al., 2014*) | ≤ 23.58 |
| ***Visuospatial, visuo-perceptive and visuo-constructive abilities*** |  |
| Benton Line Orientation Judgment Task (0-30) (*Benton et al., 1978*) | < 18 |
| Benton Facial Recognition Tests (0-54) (*Benton et al.,1983)* | < 37 |
| Rey-Osterrieth Complex Figure - Copy (0-36) (*Caffarra et al., 2002*) | < 28.88 |
| ***Social Cognition*** |  |
| Ekman 60-Faces Test (0-60) (*Dodich et al., 2014*) | ≤ 37.46 |
| Facial Complex Expressions Test (Terruzzi,Funghi et al. 2023) | ≤ 22.628 |

*S1.2* *MRI data acquisition and processing*

The MRI data were collected at the Centre for Mind/Brain Sciences (CIMeC) at the University of Trento using a 3 Tesla Siemens (Prisma) MRI scanner, with a 64-channel head receive coil. The procedure was adequately described to all participants, who were instructed to keep their eyes closed and remain awake throughout the scan. To minimise head movement during the MRI scan, the patient's head was surrounded by soft pads. The MRI scan was performed within three months of the behavioural data collection. For each participant, T1-weighted multi-echo MPRAGE structural images (van der Kouwe et al. 2008) were acquired using the following parameters: MPRAGE_GRAPPA: 176 volumes, isotropic voxel resolution of 1 mm^3^, sagittal plane orientation, flip angle of 7 degrees, matrix = 256x256, repetition time (TR) of 2.53 seconds, echo time (TE) of 1.35, 3.07, 4.79, 6.51 ms, TI of 1100 ms, slice thickness of 1 mm. Furthermore, resting-state functional MRI (rs-fMRI) was acquired using echo-planar (EPI) T2*-weighted scans with the following acquisition parameters: TE of 28 ms, TR of 1.0 seconds, flip angle of 59 degrees, axial slice thickness of 2 mm. A total of 400 whole-brain volumes were acquired in a resting-state run of six minutes and forty seconds, with isotropic voxel size of 2 mm^3^, AC/PC aligned.

*S1.3 Functional connectivity pre-processing*

Rs-fMRI analyses were performed using CONN functional connectivity toolbox (release 21.a - RRID: SCR_009550; <https://web.conn-toolbox.org>; Nieto-Castanon, 2021; Whitfield-Gabrieli & Nieto-Castanon, 2012), and Statistical Parametric Mapping (SPM12 - v7487 - RRID: SCR_007037; <https://www.fil.ion.ucl.ac.uk/spm>; Penny et al., 2011), based on MATLAB R2020b and following procedures described in a previous publication (Funghi et al. 2025). Briefly, functional and anatomical data were pre-processed using a pipeline including (a) realignment with correction of susceptibility-distortion interactions, (b) slice timing correction, (c) outlier detection, (d) direct segmentation and MNI-space normalisation, and (e) smoothing (Gaussian kernel of 8 mm full-width at half-maximum (FWHM)) only for voxel-level analyses.

*S1.4* *Grey matter pre-processing*

Anatomical images were analyzed using the Computational Anatomy Toolbox (CAT12.8.2 - r2170; [https://neuro-jena.github.io/cat](https://neuro-jena.github.io/cat/); Gaser et al., 2022) for Statistical Parametric Mapping (SPM12 - v7771; [https://www.fil.ion.ucl.ac.uk/spm](https://www.fil.ion.ucl.ac.uk/spm/);) in MATLAB R2019a as previously reported (Funghi et al. 2025). The T1-weighted anatomical images were pre-processed according to the standard VBM pipeline of CAT 12, which includes: (a) tissue segmentation into grey matter (GM), white matter (WM) and cerebrospinal fluid (CSF), (b) spatial normalization to the Montreal Neurological Institute (MNI) space, (c) modulation, and (d) spatial smoothing at 8 mm FWHM only for voxel-level analyses (Gaser et al. 2022)*.*

- 1. *Supplementary fMRI Analysis: Results of functional connectivity controlling for Age, Disease Duration, and Motor Severity*
     1. *AES-I –Nucleus Accumbens Seed*

Higher AES-I scores in PD participants were associated to significant clusters of altered functional connectivity from the right nucleus accumbens to the right putamen, paracingulate cortex and superior frontal gyrus (Table S2). No significant results were found when seeding from the left nucleus accumbens.

***Table S2. Regions with significant associations between AES-I scores and right nucleus accumbens connectivity in PD (controlling for age, disease duration, and motor severity).***

| **Peak Coordinates (x, y, z)** | **p-FDR (size)** | | **p-unc (size)** |  | |  |
| --- | --- | --- | --- | --- | --- | --- |
| -36, -84, -16 | 0.000016 | <0.000001 | | | Left - Lateral Occipital Cortex (inferior division) | |
| +28, +12, +00 | 0.0069 | 0.0002 | | | Right - Putamen | |
| +44, -66, -14 | 0.0069 | 0.0003 | | | Right - Temporal Occipital Fusiform Cortex | |
| +22, +28, +44 | 0.0228 | 0.0011 | | | Right - Superior Frontal Gyrus | |
| -14, -98, -06 | 0.0391 | 0.0027 | | | Left – Occipital Pole | |
| +10, +44, +26 | 0.0391 | 0.0029 | | | Right – Paracingulate gyrus | |

- - 1. *AES-S –Nucleus Accumbens Seed*

A significant hypoconnectivity pattern was observed between the right nucleus accumbens and the right superior parietal lobule (+14, -48, +64; p-FDR = 0.0492) associated with AES-S scores in PD participants. No significant associations were found when seeding from the left nucleus accumbens.

- - 1. *ΔAES –Nucleus Accumbens Seed*

When using the ΔAES scores as variable of interest, significant hyperconnectivity was found between the right nucleus accumbens and bilateral superior frontal gyrus (-14 +20 + 46; p-FDR = 0.001; +30 +22 + 36; p-FDR = 0.005). No significant associations were found when using the left nucleus accumbens as the seed.

- 1. *Supplementary VBM Analysis: Results controlling for Age, Disease Duration, and Motor Severity*

We conducted whole-brain VBM regression analyses considering separately AES-S, AES-I and ΔAES as covariates of interest, controlling for age, disease duration, motor severity and Total Intracranial Volume (TIV). These analyses did not reveal any FDR-corrected results. Similarly, ROI-based regression analysis showed no significant association between GM volume and either AES-S or ΔAES. We observed a significant trend between AES-I scores and the grey matter volume in the right Middle Frontal Gyrus p-_uncorrected=_ 0.05, t-value = 1.756).

**Supplementary References**

Benton, A.L. et al. 1983. *Facial Recognition: Stimulus and Multiple Choice Pictures: Contributions to Neuropsychological Assessment*. Oxford University Press, Incorporated.

Benton, A L, N R Varney, and K D Hamsher. 1978. “Visuospatial Judgment. A Clinical Test.” *Archives of neurology* 35(6): 364–67.

Caffarra, P. et al. 2002a. “Rey-Osterrieth Complex Figure: Normative Values in an Italian Population Sample.” *Neurological Sciences* 22(6): 443–47.

———. 2002b. “Una Versione Abbreviata Del Test Di Stroop: Dati Normativi Nella Popolazione Italiana.” *Rivista di Neurologia* 12(4): 111–15.

Carlesimo, G A et al. 1996. “The Mental Deterioration Battery: Normative Data, Diagnostic Reliability and Qualitative Analyses of Cognitive Impairment.” *European Neurology* 36(6): 378–84.

Catricalà, Eleonora et al. 2013. “An Italian Battery for the Assessment of Semantic Memory Disorders.” *Neurological Sciences* 34(6): 985–93.

Dodich, Alessandra et al. 2014. “Emotion Recognition from Facial Expressions: A Normative Study of the Ekman 60-Faces Test in the Italian Population.” *Neurological Sciences* 35(7): 1015–21.

Funghi, Giulia et al. 2025. “Mental State Recognition Deficits Linked to Brain Changes in Parkinson’s Disease Without Dementia.” *European Journal of Neuroscience* 61(4): e70014.

Gaser, Christian et al. 2022. Biorxiv *CAT – A Computational Anatomy Toolbox for the Analysis of Structural MRI*.

Giovagnoli, A R et al. 1996. “Trail Making Test: Normative 287 Normal Adult Controls.” *Italian journal of neurological sciences* 17(4): 305–9. https://doi.org/10.1007/BF01997792.

van der Kouwe, André J.W., Thomas Benner, David H. Salat, and Bruce Fischl. 2008. “Brain Morphometry with Multiecho MPRAGE.” *NeuroImage* 40(2): 559–69.

Litvan, Irene et al. 2012. “Diagnostic Criteria for Mild Cognitive Impairment in Parkinson’s Disease: Movement Disorder Society Task Force Guidelines.” *Movement Disorders* 27(3): 349–56.

Monaco, Marco, Alberto Costa, Carlo Caltagirone, and Giovanni Augusto Carlesimo. 2013. “Forward and Backward Span for Verbal and Visuo-Spatial Data : Standardization and Normative Data from an Italian Adult Population.” : 749–54.

Nieto-Castanon, Alfonso. 2021. “CONN Functional Connectivity Toolbox (RRID: SCR_009550), Version 21.”

Papagno, Costanza, Alessandra Casarotti, Barbara Zarino, and Davide Crepaldi. 2020. “A New Test of Action Verb Naming: Normative Data from 290 Italian Adults.” *Neurological Sciences* 41(10): 2811–17.

Penny, W. D. et al. 2011. *Statistical Parametric Mapping: The Analysis of Functional Brain Images*. ed. Elsevier.

Spinnler, H, and G Tognoni. 1987. “Standardizzazione e Taratura Italiana Di Test Neuropsicologici.” *Italian journal of neurological sciences* Suppl. 8.

Terruzzi, Stefano et al. 2023. “The FACE Test: A New Neuropsychological Task to Assess the Recognition of Complex Mental States from Faces.” *Neurological Sciences* 44(7): 2339–47. https://doi.org/10.1007/s10072-023-06697-w.

Whitfield-Gabrieli, Susan, and Alfonso Nieto-Castanon. 2012. “Conn: A Functional Connectivity Toolbox for Correlated and Anticorrelated Brain Networks.” *Brain connectivity* 2(3): 125–41.

Zarino, Barbara, Marta Crespi, Michela Launi, and Alessandra Casarotti. 2014. “A New Standardization of Semantic Verbal Fluency Test.” *Neurological Sciences* 35(9): 1405–11.
